# Supplementary material for: Structural mechanisms of phospholipid activation of the human TPC2 channel
Source: eLife. 2019 Mar 12;8:e45222. doi: 10.7554/eLife.45222 (PMC6424560; doi:10.7554/eLife.45222)
Supplement: Figure 1—source data 1. [file elife-45222-fig1-data1.docx]

**Figure 1—source data 1**

**Cryo-EM data collection and model statistics**

|  | Ligand-bound  open HsTPC2  (EMD_0477)  (PDB 6NQ0) | Ligand-bound closed HsTPC2  (EMD_0479)  (PDB 6NQ2) | Apo HsTPC2  (EMD_0478)  (PDB 6NQ1) |
| --- | --- | --- | --- |
| **Data collection and processing** |  |  |  |
| Magnification | 46730 | 46730 | 46730 |
| Voltage (kV) | 300 | 300 | 300 |
| Electron exposure (e–/Å^2^) | ~50 | ~50 | ~50 |
| Defocus range (μm) | -1.5 to -3.0 | -1.5 to -3.0 | -1.5 to -3.0 |
| Pixel size (Å) | 1.07 | 1.07 | 1.07 |
| Symmetry imposed | *C2* | *C2* | *C2* |
| Initial particle images (no.) | 653,430 | 653,430 | 227, 926 |
| Final particle images (no.) | 33,441 | 56,548 | 96,361 |
| Map resolution (Å)  FSC threshold | 3.7  0.143 | 3.4  0.143 | 3.5  0.143 |
|  |  |  |  |
| **Refinement** |  |  |  |
| Initial model used (PDB code) | 6C9A | 6C9A | 6C9A |
| Model resolution (Å)  FSC threshold | 3.7  0.143 | 3.4  0.143 | 3.5  0.143 |
| Map sharpening *B* factor (Å^2^) | -121.09 | -125.71 | -112.67 |
| Model composition  Non-hydrogen atoms  Protein residues  Ligands | 10140  10046  94 | 10140  10046  94 | 10046  10046  0 |
| *B* factors (Å^2^)  Protein  Ligand | 95.42  55.02 | 73.41  55.02 | 84.09  N/A |
| R.m.s. deviations  Bond lengths (Å)  Bond angles (°) | 0.005  0.887 | 0.009  0.940 | 0.008  0.966 |
| Validation  MolProbity score  Clashscore  Poor rotamers (%) | 1.45  3.57  0.36 | 1.48  3.42  0.36 | 1.52  3.69  0.18 |
| Ramachandran plot  Favored (%)  Allowed (%)  Disallowed (%) | 95.58  4.42  0 | 94.93  5.07  0 | 94.76  5.24  0 |
