# Supplementary material for: Structural mechanisms of phospholipid activation of the human TPC2 channel
Source: eLife. 2019 Mar 12;8:e45222. doi: 10.7554/eLife.45222 (PMC6424560; doi:10.7554/eLife.45222)
Supplement: Figure 4—source data 1. [file elife-45222-fig4-data1.pdf]

Source Data for Figure 4A

[I/I_{Max} measured at -100 mV with various PI(3,5)P2 concentrations, where I_{Max} is the current at 30uM PI(3,5)P2 .]

3,5-PIP2 in uM	#1	#2	#3	#4	#5	Mean	SEM
0.03	0.0124	0.0063	0.0300	0.0300	0.0135	0.0184	0.0049
0.1	0.0592	0.0627	0.1943	0.1800	0.0767	0.1146	0.0299
1	0.7262	0.7114	0.6986	0.7057	0.8819	0.7448	0.0346
10	0.9199	0.9227	0.9314	0.9743	0.9577	0.9412	0.0106
30	1	1	1	1	1	1	0

Source Data for Figure 4B

[Normalized currents of HsTPC2 measured at -100 mV with various phosphatidylinositol lipids at 10 µM.]

	#1	#2	#3	#4	#5	#6	Mean	SEM
3,5-PIP2	1	1	1	1	1	1	1	0
4,5-PIP2	0.0603	0.0691	0.1053	0.0333	0.0337	0.0100	0.0520	0.0150
3,4-PIP2	0.0171	0.0306	0.0147	0.0269	0.0274	0.0083	0.0209	0.0039
5-PIP2	0.0207	0.0309	0.0368	0.03	0.0141	0.0130	0.0243	0.0044
3-PIP2	0.0362	0.037	0.0408	0.0166	0.0234	0.0337	0.0313	0.0042

Source Data for Figure 4E
[Current density (pA/pF) at -100 mV]

	TPC2 (WT)	K203A	K204A	K207A	S322A	R329A
#1	132.4	9.8	12.6	123.5	54.1	18.2
#2	315.8	20.6	18.2	63.2	65.4	15.6
#3	253.6	11.8	8.9	78.6	120.6	21.2
#4	180.8	31.7	7.9	45.6	140.8	19.8
#5	156.2	13.6	12.8	60.2	154.4	32.6
#6	276.3	35.6	15.6	50.4	45.3	35.7
#7	217.9	15.6	14.8	110.5		27.8
#8	364.7	17.8	9.7	135.6		42.8
#9	259.3	8.2	13.5	52.3		43.5
Mean	239.7	18.3	12.7	80.0	96.8	28.6
SEM	25.2	3.2	1.1	11.4	19.4	3.5

3,5-PIP2 dose-response for mutants shown in Figure 4F

[I/I_{Max} measured at -100 mV with various PI(3,5)P2 concentrations, where I_{Max} is the current at 100uM PI(3,5)P2 .]

TPC2_K203A

PIP2(uM)	#1	#2	#3	#4	#5	Mean	SEM
1	0.0512	0.0273	0.0095	0.0415	0.0235	0.0306	0.0072
3	0.1025	0.0909	0.0238	0.0824	0.0588	0.0717	0.0140
10	0.2011	0.2727	0.4048	0.3201	0.2353	0.2868	0.0355
30	0.7829	0.9091	0.9048	0.7906	0.8235	0.8422	0.0273
100	1	1	1	1	1	1	0

TPC2_K204A

PIP2(uM)	#1	#2	#3	#4	#5	Mean	SEM
1	0	0.025	0	0	0.1251	0.0300	0.0242
3	0	0.1054	0.0941	0.2222	0.2502	0.1344	0.0456
10	0.4211	0.3106	0.3824	0.4444	0.6254	0.4368	0.0523
30	0.9211	0.8959	0.9412	0.7778	0.8755	0.8823	0.0284
100	1	1	1	1	1	1	0

TPC2_K207A

PIP2(uM)	#1	#2	#3	#4	#5	Mean	SEM
0.1	0	0	0	0	0	0	0
1	0.0632	0.0630	0.0719	0.0458	0.0743	0.0636	0.0050
3	0.3150	0.4590	0.2758	0.4730	0.2340	0.3514	0.0486
10	0.7895	0.8148	0.7994	0.9160	0.7921	0.8224	0.0238
30	0.9474	0.9630	0.9592	0.9924	0.9406	0.9605	0.0089
100	1	1	1	1	1	1	0

3,5-PIP2 dose-response for mutants shown in Figure 4F

[I/I_{Max} measured at -100 mV with various PI(3,5)P2 concentrations, where I_{Max} is the current at 100uM PI(3,5)P2 .]

TPC2_S322A

PIP2(uM)	#1	#2	#3	#4	#5	#6	Mean	SEM
0.1	0	0	0	0	0	0	0	0
1	0.0286	0.1429	0.1038	0.0593	0.0368	0.0968	0.0780	0.0180
3	0.2857	0.7810	0.6538	0.7407	0.4211	0.6452	0.5879	0.0790
10	0.8286	0.9143	0.8846	0.9259	0.8632	0.8065	0.8705	0.0192
30	0.9714	0.9524	0.9615	0.9630	0.9684	0.9677	0.9641	0.0028
100	1	1	1	1	1	1	1	0

TPC2_R329A

PIP2(uM)	#1	#2	#3	#4	#5	Mean	SEM
0.1	0	0	0	0	0	0	0
1	0.0444	0.0231	0.0352	0.0354	0.0565	0.0389	0.0055
3	0.1900	0.2800	0.2100	0.3500	0.1200	0.2300	0.0394
10	0.6222	0.7077	0.7042	0.7080	0.7609	0.7006	0.0223
30	0.8889	0.9385	0.9507	0.9292	0.9239	0.9262	0.0104
100	1	1	1	1	1	1	0
